# Study of {332}<113> twinning in a multilayered Ti-10Mo-xFe (x = 1–3) alloy by ECCI and EBSD

**DOI:** 10.1080/14686996.2016.1177439

**Published:** 2016-05-16

**Authors:** Ivan Gutierrez-Urrutia, Cheng-Lin Li, Satoshi Emura, Xiaohua Min, Koichi Tsuchiya

**Affiliations:** ^a^ National Institute for Materials Science, Tsukuba, Ibaraki305-0047, Japan; ^b^ School of Materials Science and Engineering, Dalian University of Technology, Dalian116024, PR China; ^c^ Graduate School of Pure and Applied Sciences, University of Tsukuba, Ibaraki305-0047, Japan

**Keywords:** Dislocations, plasticity, interface, multi-layered materials, 10 Engineering and Structural materials, 106 Metallic materials, 100 Materials, 303 Mechanical / Physical processing, 300 Processing / Synthesis and Recycling, 503 TEM, STEM, SEM, 500 Characterization

## Abstract

We have investigated the propagation of {332}<113> twins in a multilayered Ti-10Mo-xFe (x = 1–3) alloy fabricated by multi-pass hot rolling. The material contains a macroscopic Fe-graded structure (about 130 μm width) between 1 and 3 wt% Fe in the direction perpendicular to rolling. We observe strong influence of the Fe-graded structure in the twin propagation behavior. The propagation of {332}<113> twins that are nucleated in Fe-lean regions (~1 wt% Fe) is interrupted in the grain interiors at a specific Fe content, namely, about 2 wt% Fe. We ascribe this effect to the role of Fe content in solid solution on the stress for twin propagation. The interruption of twins in the grain interiors results in the development of characteristic dislocation configurations such as highly dense dislocation walls (HDDWs) associated to strain localization phenomena. The nucleation and propagation of these dislocation configurations is ascribed to the underlying plastic accommodation mechanisms of the stress field at the twin tips. We find that the crystallographic alignment of HDDWs is determined by the stress field at the twin tips and the deformation texture. The excellent plastic accommodation at the interrupted twin tips allows attaining the good ductility of the present material (total elongation of 28%).

## Graphical abstract

**Figure:**
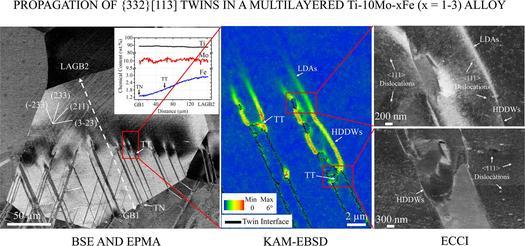

## 1. Introduction

In the last decade, β-Ti-Mo alloys have received considerable attraction as structural materials in several technological areas such as aerospace, automotive and biomedical industries. These alloys contain outstanding combination of high specific strength, low elastic modulus and excellent bio-compatibility [1–6] Several processing techniques such as rolling, drawing and forging are typically used to optimize the mechanical behavior of these alloys [1–6] Recently, Min et al. [7] have processed a multilayered β-Ti-10Mo-xFe (x = 1–3 wt%) alloy by multi-pass hot rolling that exhibits superior combination of strength and ductility (yield strength, YS, of 800 MPa; total elongation, TE, of 28%), compared to those of the individual alloys. This material contains a macroscopic gradient in Fe that tailors the combination of specific deformation mechanisms, namely, <111> dislocation slip and {332}<113> deformation twinning that results in superior mechanical behavior.

The activation of deformation modes in β-Ti-Mo alloys is associated to the stability of β-Ti (bcc phase) against the β-α′ and β-α′′ stress-induced phase transformation [8–11] Whereas <111> slip has been intensively studied in bcc metals [12–15] the details of {332}<113> twinning mode are still unclear [16–18] Specifically, two main models have been proposed for {332}<113> twinning, namely, shear and shuffle-type mechanisms,[16,17,19] and a pole dislocation-type model involving partial dislocations.[18,20] The first approaches suggest that twinning takes place by a complex atomic movement that involves atomic shearing and shuffling by specific crystallographic criteria. The dislocation-type model suggests that <111> perfect dislocations are dissociated into <113> dislocations after characteristic dislocation interactions. The subsequent revolution of <113> dislocations around the dislocation node by the pole mechanism results in the growth of the twin plate. The twin models described above provide relevant details on the crystallography and nucleation mechanism of {332}<113> twinning. However, twin propagation also plays significant role on the strain hardening behavior and fracture mode of polycrystalline alloys. The influence of microstructure on twin propagation is complex mainly due to the large number of parameters involved.[21–30] In particular, several works have addressed the influence of grain size [21,27,29,30] and twin–twin interactions [25] on twin propagation in several bcc alloys.

Novel β-Ti-Mo alloys such as multilayered materials contain another interesting microstructural feature that may also influence twin propagation phenomena, namely, chemical graded structure. The propagation of a twin plate through a composition gradient may be different to that in homogeneous alloys due to solute effects on twinning mode [16,17] and twin propagation stress,[21,29] which are currently unclear. The aim of this work is to investigate the propagation of {332}<113> deformation twins in a multilayered β-Ti-10Mo-xFe (x = 1–3) alloy that contains a macroscopic composition gradient of Fe. Microstructure characterization was performed by electron probe microanalysis (EPMA), electron backscatter diffraction (EBSD) and electron channeling contrast imaging (ECCI). The material was fabricated by multi-pass hot rolling and contains a Fe-graded structure ranged between 1 and 3 wt% of about 130 μm width. We observe a strong influence of the Fe-graded structure in the twin propagation behavior. Specifically, the propagation of {332}<113> twins is interrupted at a certain Fe content, namely, about 2 wt% Fe. We ascribe this effect to the role of Fe content on the stress for twin propagation. The occurrence of interrupted growth twins in the grain interiors results in the development of specific dislocation configurations that are ascribed to the underlying plastic accommodation mechanism at the twin tips. We claim that this effect contributes to the good ductility of the multilayered Ti-Mo-Fe alloy (total elongation of 28%).

## 2. Experimental details

A multi-layered stacked material consisting of 10 alternative layers of Ti-10Mo-1Fe and Ti-10Mo-3Fe alloys (wt%) was used in this study. To generate a macroscopic Fe-graded structure, the material was processed by multi-pass hot rolling at 1000 °C and subsequent solution treatment at 900°C for 1 h.[7] According to the current melting method (cold crucible levitation melting) and thermomechanical conditions, the oxygen content is negligible (<0.1 wt%).[31] Tensile samples were machined out from the multilayered material and from individual alloys, namely, Ti-10Mo-1Fe, Ti-10Mo-2Fe and Ti-10Mo-3Fe. The tensile samples had 18 mm gage length, 4 mm gage width and 2.5 mm gage thickness. Tensile axis was parallel to the rolling direction (RD). Complete and interrupted tensile tests to a true strain of 4% were performed on an Instron 5581 tensile machine at room temperature and at an initial strain rate of 2.8×10^−4^ s^−1^. Deformation structure was characterized by two Scanning Electron Microscopy (SEM) techniques, namely ECCI and EBSD in a Sigma Zeiss field emission gun scanning electron microscope (Carl Zeiss AG, Oberkochen, Germany)(FEG-SEM) equipped with a TSL (TSL, EDAX, NJ, USA) Orientation Imaging Microscopy (OIM) EBSD system. The rolling direction (RD)–normal direction (ND) section of the tensile sample was evaluated. High-resolution EBSD maps with a step size of 50 nm were taken at 15 kV acceleration voltage. The deformation structure of the grains mapped by EBSD was subsequently examined by ECCI under controlled diffraction conditions as described in [32,33]. ECCI images were obtained with optimum channeling contrast by tilting the matrix crystal into the Bragg condition in a “two-beam” mode using a high intensity diffraction vector g_111_. ECCI observations were carried out at a working distance of 4–5 mm and at 9 kV acceleration voltage by using an objective aperture of 120 μm. Elemental mapping was performed by EPMA on a JEOL JXA-8900F system operated at 15 kV.

## 3. Results 

**Figure 1.**
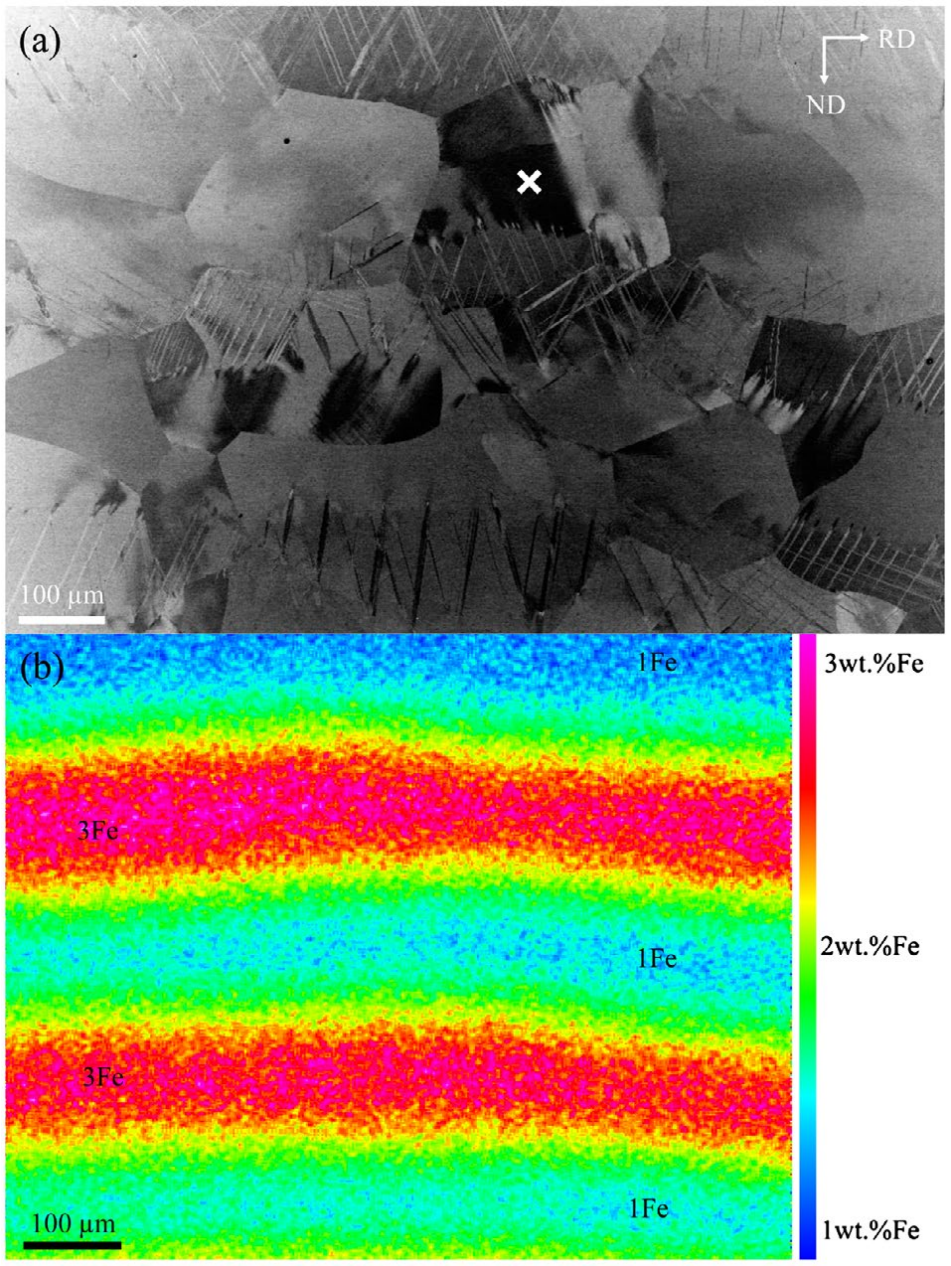
(a) ECCI image of the deformation structure of the multilayered Ti-10Mo-xFe (x = 1–3) material tensile deformed to 0.04 true strain. The white cross indicates the grain analyzed in Figure 2. (b) EPMA map of Fe distribution of the grain indicated by the white cross in (a).

Figure 1 shows the deformation microstructure (a) and the elemental distribution (b) of the multilayered material tensile deformed to 0.04 true strain. The elemental map shows that at the present thermomechanical conditions, the material contains a macroscopic gradient of Fe ranged between 1 and 3 wt% Fe that is distributed in the form of bands parallel to RD, while Ti and Mo are homogeneously distributed within the Fe-graded structure [7]. These bands are about 130 μm thick (green to yellow to red colored bands in Figure 1(b)) and are stacked between the original Ti-10Mo-1Fe and Ti-10Mo-3Fe layers, which are about 50 μm thick (3 wt% Fe: pink layer; 1 wt% Fe: blue layer). The width of the Fe-graded structure is determined by the thermomechanical conditions. At the present conditions and considering the diffusion coefficient of Fe in β-Ti (60 × 10^−13^ m^2^ s^−1^),[34] a diffusion length of Fe in β-Ti of about 50 μm is obtained. Using these data, the estimated length of the Fe concentration profile between two layers is about 100 μm, which roughly agrees with the experimental value of 130 μm. Figure 1(a) shows that the deformation structure is formed by {332}<113> deformation twins and <111> slip traces. This structure is similar to that reported in conventional β-Ti-Mo alloys.[14,35–38] However, in the present multilayered Ti-Mo-Fe alloy the propagation of deformation twins is interrupted within the grain interiors at the same location, i.e. at about 2 wt% Fe. This effect results in the confinement of twin plates within areas of about 100 μm long (average grain size is about 250 μm). This finding indicates that the Fe-chemical graded structure has a significant influence on the propagation of {332}<113> deformation twins in the present Ti-Mo-Fe alloy.

**Figure 2.**
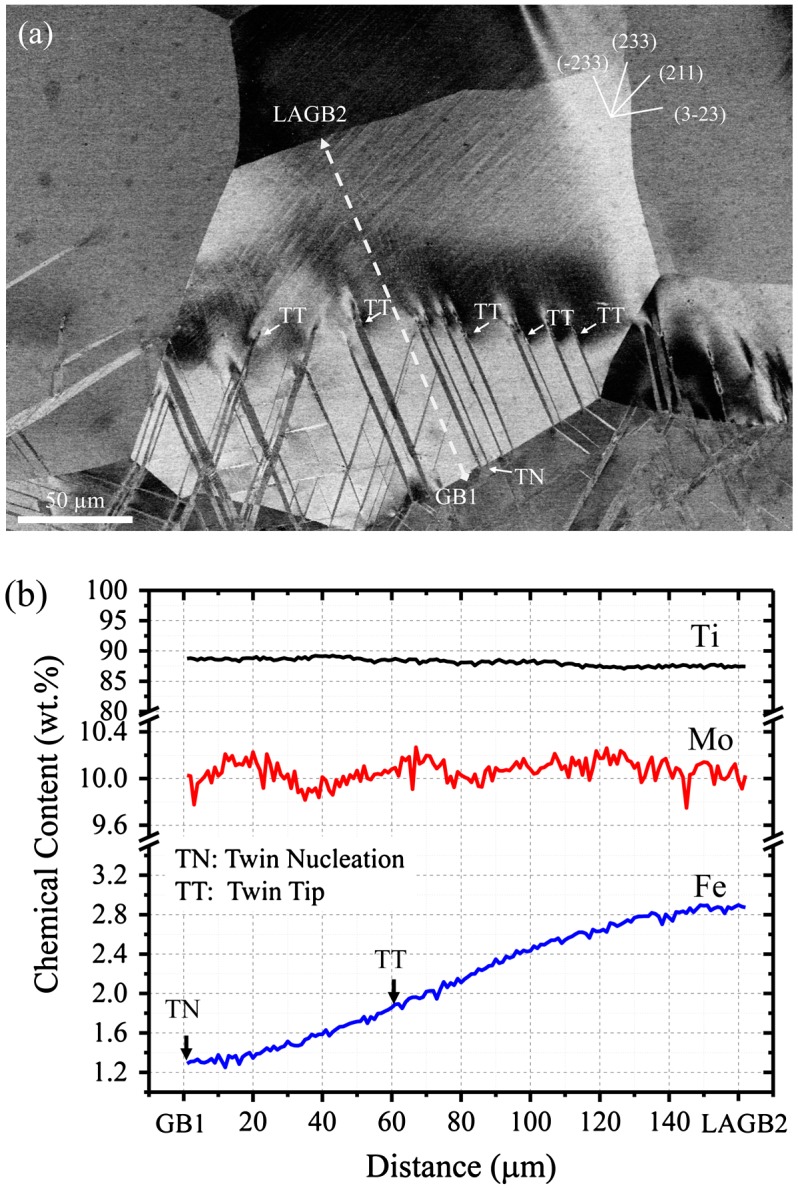
(a) ECCI image of the deformation structure of a typical grain. The grain is oriented close to the [1 15 16] crystallographic orientation. (b) Chemical line analysis performed by EPMA along the dashed line of the grain shown in (a). TN: twin nucleation; TT: twin tip; GB: grain boundary; LAGB: low angle grain boundary.

Figure 2(a) shows an example of the deformation structure of a grain oriented close to the [1 15 16] crystallographic direction (the location of the grain is indicated by the cross in Figure 1(a)). This ECCI image was obtained by tilting the sample in the channeling condition with g_111_ excited so that the crystal matrix is in an out-of-Bragg condition and hence it appears as bright background, whereas the twin crystal plates are close to the Bragg condition and accordingly, they appear dark. The grain contains two active twin systems that correspond to the twin systems with the highest Schmid factors (SFs), namely, (−233)[311] with SF = 0.41, and (233)[−311] with SF = 0.36. These twins are nucleated at GB1 (TN: twin nucleation in Figure 2(a)) and are interrupted at an area containing about 1.8 wt% Fe (TT: twin tip in Figure 2(a)), i.e. they propagate through the grain interior a finite length of about 60 μm. Elemental mapping reveals that the grain contains a Fe gradient of about of about 0.01 wt% Fe/μm which is 160 μm long. Specifically, Fe content varies between 1.2 wt% at the grain boundary GB1 and 2.8 wt% at the low angle grain boundary LAGB2. On the other hand, Mo content is relatively homogeneous and ranges between 9.8 and 10.2 wt%. Detailed analysis of the EPMA data, Figure 2(b), did not reveal the occurrence of any chemical interface within the composition gradient (line analysis along the dashed line in Figure 2(a)).

**Figure 3.**
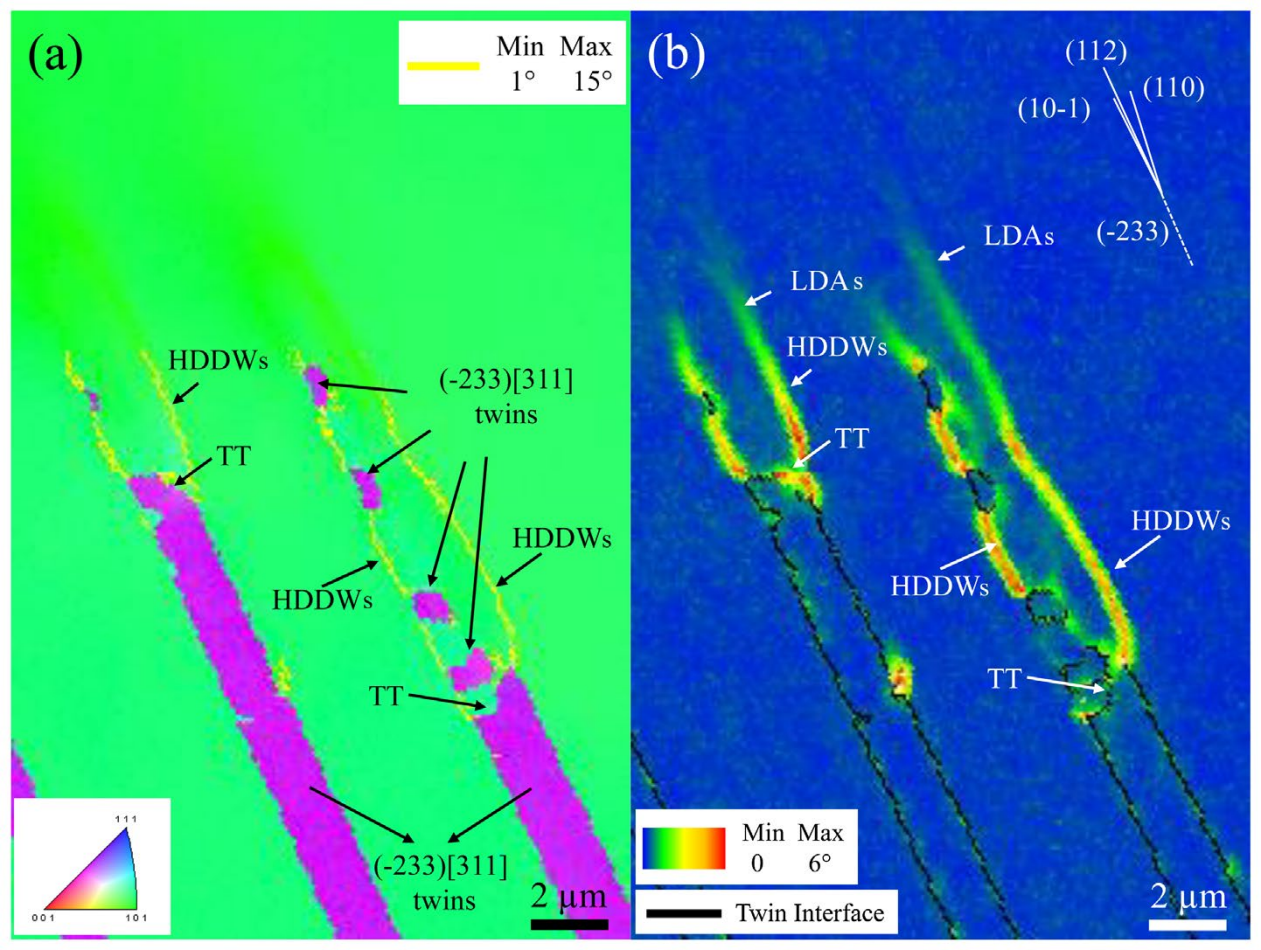
EBSD maps of the deformation structure around two interrupted twins inside the grain shown in Figure 2(a). (a) IPF-EBSD map along the tensile direction. Yellow line represents low angle grain boundaries (misorientation between 1 and 15°); (b) KAM-EBSD map. Black line indicates twin interfaces. HDDWs: highly dense dislocation wall; LDAs: loose dislocation arrangements; TT: twin tip.

**Figure 4.**
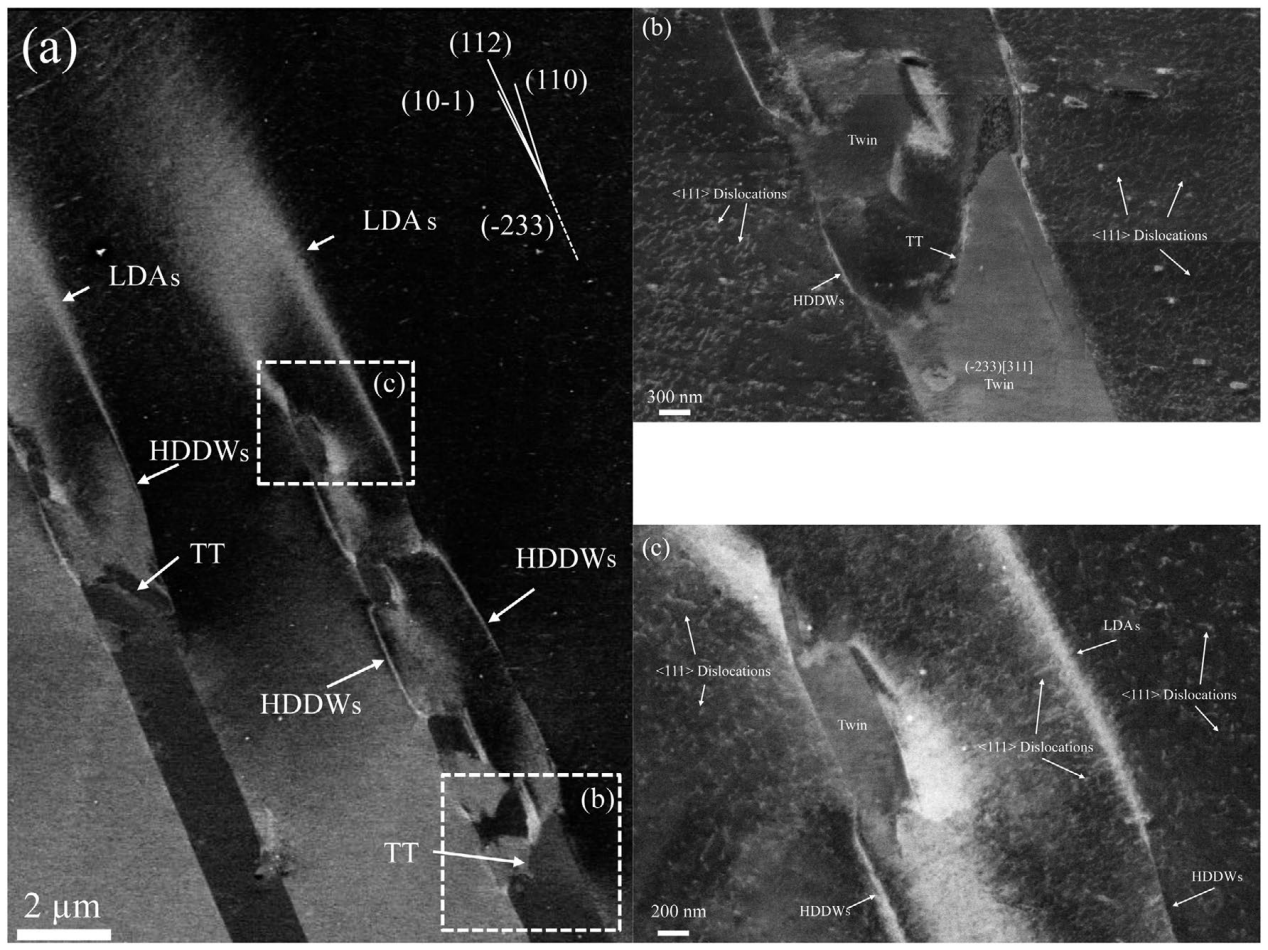
ECCI images of the deformation structure of the twins shown in Figure 3. Images were taken with g_111_ excited close to Bragg condition. (b) and (c) reveal details of the dislocation configuration of the areas indicated by dashed boxes in (a). HDDWs: highly dense dislocation wall; LDAs: loose dislocation arrangements.TT: twin tip.

Figures 3 and 4 show the deformation structure around two interrupted twin tips of the grain shown in Figure 2. Specifically, Figures 3(a) and (b) correspond to an IPF-EBSD map along the tensile direction and a kernel average misorientation (KAM)-EBSD map (first neighbors), respectively. These EBSD maps reveal that the deformation structure is mainly formed by paired dislocation layers (indicated by yellow lines in Figure 3(a)) and few small twin plates of about 1 μm long and 0.5 μm width. The twin system of these small twins is the same as that of the primary twins, namely, (−233) [311]. The dislocation layers are nucleated at the edges of the twin tips (TT in Figures 3 and 4) and propagate into the matrix for a length of about 5 μm. The KAM-EBSD map of Figure 3(b) shows that these dislocation configurations are misoriented between 3 and 15° from the crystal matrix. The misorientation angle *θ* decreases with the distance from the twin tip, namely, from *θ* = 10–15° in the vicinity of the twin tips to *θ* ~ 3° at 5 μm from the twin tips.

Details of the dislocation configurations around the twin tips are revealed by the ECCI images of Figure 4, which were taken with g_111_ excited close to Bragg condition. Channeling contrast of dislocation configurations reveals that the paired dislocation layers correspond to highly dense dislocation walls (HDDWs).[39,40] These dislocation configurations are imaged by ECCI at the current channeling conditions as dense dislocation arrays which appear as bright straight compact layers; see Figure 4(b). HDDWs are characteristic of fcc metals with pronounced planar slip such as Al-Mg, Fe-Mn-C and Fe-Mn-Al-C alloys.[39,41,42] We observe that HDDWs tend to align almost parallel to the (−233) twin habit plane trace; see Figure 4(a). Specifically, they lay along three slip systems, namely, (112), (10–1) and (110), which are slightly deviated from the (−233) twin habit plane (about 2–5°). The KAM-EBSD map of Figure 3(b) reveals that HDDWs are formed by a high density of geometrically necessary dislocations (GNDs) that enables the accommodation of matrix misorientations of up to 15° in the vicinity of the twin tips. The dislocation density of HDDWs is not uniform, as reflected in the decrease of the misorientation angle (*θ*) with the distance from the twin tip. The propagation of HDDWs in the matrix is limited to a distance of about 2–5 μm. At this point, ECCI reveals that the dislocation substructure evolves from planar configurations to loose dislocation arrangements (LDAs) that propagate about 5 μm further into the matrix; see Figure 4(c). These dislocation configurations are imaged by ECCI at the current channeling conditions as wide loose bright layers. They are visualized in the corresponding KAM map (Figure 3(b)) as localized orientation gradients with *θ* ~ 1–3°. Figure 4(c) also reveals that the interior of the crystal delimited by HDDWs/LDAs contains high density of <111>-type dislocations gliding on {110} and {112} slip planes (dislocations are imaged in Figure 4(c) as bright sharp lines over a dark matrix background).

## 4. Discussion 

### 4.1. Propagation of twins 

Homophase interfaces such as grain boundaries, annealing twins, and mechanical twins play a significant role on the propagation of mechanical twins in bcc metals.[21,29,43] In particular, grain boundaries and twin interfaces can act as strong barriers on twin propagation and, depending on the crystallographic orientation of the crystals involved, may even completely hinder twin propagation. The propagation of twins in the present chemical graded Ti-10Mo-xFe (x = 1–3) alloy is interrupted in the grain interiors at a specific Fe content, namely, about 2 wt% Fe. EPMA and high-resolution EBSD mapping do not reveal the formation of any intragranular homophase interface such as diffuse chemical interface, low angle grain boundary or annealing twin. On the other hand, in the present Ti-Mo-Fe alloy and under the current thermomechanical conditions, the role of precipitates such as ω-omega phase on the precipitate-twin interaction can be ruled out due to the small volume fraction (<0.01) and their shearable nature.[15,44] These findings indicate that Fe content in solid solution is the main microstructural parameter controlling twin propagation in the present chemical graded Ti-Mo-Fe alloy.

**Figure 5.**
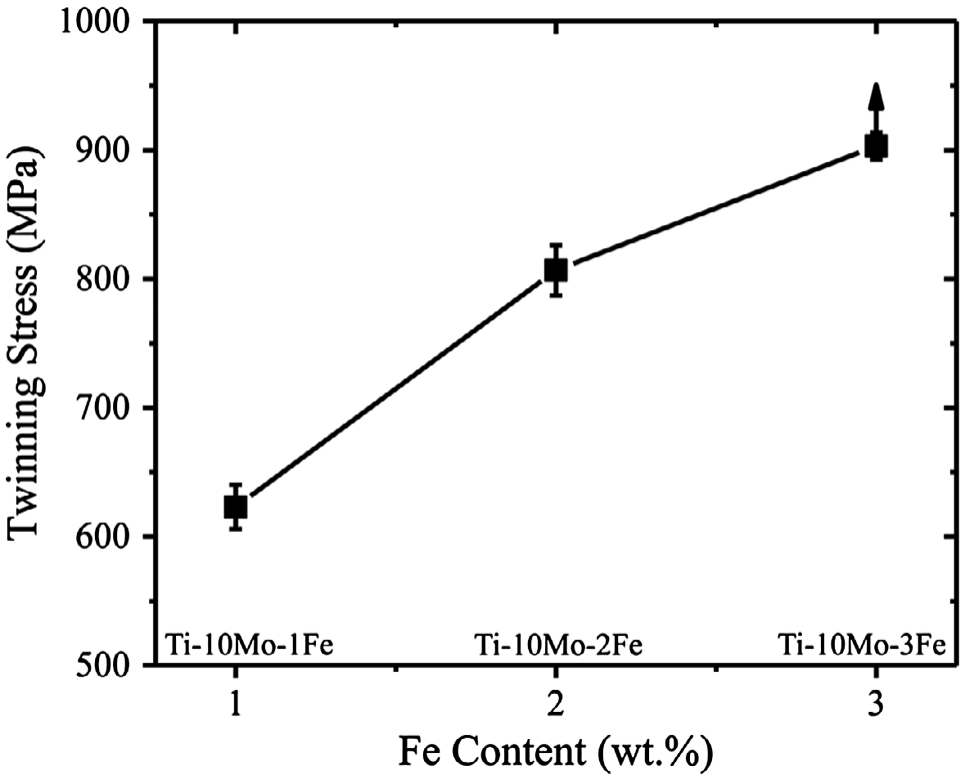
Effect of the Fe content on the twinning stress in the Ti-10Mo alloy system. Error bars indicate experimental deviation.

In order to clarify the role of Fe content on twin propagation, we have estimated the twinning stress, *σ*_*tw*_, of the Ti-10Mo-xFe (x = 1–3) system. We define *σ*_*tw*_ as the macroscopic stress at which twins are detected. According to dislocation-based models of twinning in bcc metals,[21,29,30,43] the experimental twinning stress corresponds to the stress to grow an existing twin nuclei. Specifically, the twinning stress of three alloys, namely Ti-10Mo-1Fe, Ti-10Mo-2Fe and Ti-10Mo-3Fe, was determined by tensile loading experiments. Deformation structure was examined by ECCI and EBSD. The alloys contained similar average grain sizes of ~250 μm in order to rule out grain size effects on twinning stress. The alloys with low Fe content, i.e. Ti-10Mo-1Fe and Ti-10Mo-2Fe, contained {332}<113> twins readily at yielding. For these alloys we thus consider twin stress as the yield stress. On the other hand, deformation twins were not visible in the Ti-10Mo-3Fe alloy at any investigated strain level. Accordingly, we can only estimate a lower bound of the twinning stress for this alloy system. The experimental values of the twin stress with respect to Fe content are plotted in Figure 5. This figure shows that twinning stress scales with Fe content. We observe that additions of Fe higher than 1 wt% promote a significant increase of the twinning stress, namely, 2 wt% Fe increases the twinning stress about 205 MPa, and 3 wt% Fe increases the twinning stress at least 330 MPa. These findings indicate that the stress to propagate a twin plate that is nucleated in the Fe-lean region (~1 wt% Fe) and propagates through the Fe-graded structure increases significantly with the twin propagation length. At the current deformation conditions, the macroscopic applied stress is about 830 MPa, which is slightly higher than the twinning stress for the Ti-10Mo-2Fe alloy (*σ*_*tw*_ = 780 MPa). This result suggests that twins nucleated in the Fe-lean region (~1 wt% Fe) are able to propagate within the interior of a grain up to an area containing about 2 wt% Fe, which is close to the experimental value of 1.8 wt% Fe, see Figure 2(b). This difference can be explained as follows. The twinning stress is given by two contributions, namely, one term that is associated to the critical resolved shear stress (*τ*_*critical*_) to grow a twin, and another term (*τ*_*GS*_) that accounts for the role of grain boundaries on twin propagation,[21,29,30,43] i.e.:

[1] $$s_{tw}=M\left(t_{critical}+t_{GS}\right)$$

where *M* is the Taylor factor. *τ*_*critical*_ is determined by the corresponding twinning model. Its mathematical expression is still unknown. *τ*_*GS*_ is commonly considered as a Hall–Petch type relation (*τ*_*GS*_ *= K*/*D* ^1/2^) or Frank–Read type stress (*τ*_*GS*_ *= Gb*/*D*), where *K* is a constant, *D* is the average grain size, *G* is the shear modulus and *b* is the magnitude of Burgers vector.[21,23,29,30] The experimental twinning stress values of Figure 5 were obtained in polycrystalline alloys. As Eq. (1) shows, grain boundary effects on twin propagation (*τ*_*GS*_) are inherently estimated in such experiments. However, these effects are not relevant for the present case where we investigate the propagation of twins within a grain interior free of interfaces, similar to that occurring in single crystals. Accordingly, we envisage that the calculated twin propagation lengths by using *σ*_*tw*_ of individual polycrystalline alloys are slightly overestimated, which agrees with our findings.

### 4.2. Plastic accommodation of twin tips

The main relevant feature of the deformation structure associated to the plastic accommodation of the twin tips is the formation of specific dislocation configurations such as HDDWs and LDAs around the twin tips. HDDWs are characteristic of strain localization phenomena associated to intense dislocation activity on single slip or texture softening.[39,45–48] They are formed along highly stressed crystallographic planes and typically contain high density of GNDs. In the present case, we observe that HDDWs are nucleated at the edges of the twin tips and propagate into the matrix for about 2–5 μm. At this point, HDDWs evolve into loose dislocation arrangements, i.e. they lose their planar confinement and become less defined dislocation configurations.

The formation of HDDWs at the edges of the twin tips suggests that these locations contain high stress concentrations, and hence are the main sources of plasticity. This observation agrees with the geometry of the stress field around twin tips calculated by the dislocation theory.[49–53] In particular, the grain analyzed here, which is oriented close to the [1 15 16] crystallographic orientation, contains three slip planes where HDDWs are propagated, namely, (112), (10–1) and (110). These slip planes are slightly deviated to the trace of the (−233) twin habit plane (~2–5°). As these slip planes do not follow the Schmid law with respect to the macroscopic tensile stress state, we suggest that these planes correspond to the highly stressed slip planes with respect to the microscopic stress field around the twin tip.

The evolution of HDDWs into loose dislocation arrangements can be explained as follows. ECCI images of the deformation structure around the twin tips show a high density of <111>-type dislocations within the crystal delimited by the paired HDDWs; see Figures 4(b) and (c). Slip trace analysis reveals that they glide on {112} and {011} slip planes. In the current grain, <111> pencil glide rotates the tensile axis toward [11−1] direction [54]. This crystal rotation can be described as a single rotation around the [101] axis. This means that the intense dislocation activity around the twin tip results in lattice rotations mainly around [101] axis. As a result, the crystal delimited by the paired HDDWs is just rotated several degrees (5–15° from EBSD) around [101] axis with respect to the crystal matrix away from HDDWs. This finding indicates that HDDWs can be considered as simple dislocation tilt boundaries. The stable configuration of dislocation tilt boundaries requires a specific dislocation spacing that is determined by the local stress field.[52,53] Perturbations of the stress field may destabilize such tilt boundaries that subsequently evolve into less defined arrangements. We suggest that the evolution of well-defined dislocation arrangements such as HDDWs to LDAs is associated to the decrease of the intensity of the stress field with the distance from the twin tip. It is worth commenting that due to the bcc structure, the grains with stable crystallographic orientations after tensile loading (orientations along the line between <100>//tensile axis and <110>//tensile axis) contain several {110} and {112} crystallographic slip planes that are slighted deviated from the {233} twin habit planes. Accordingly, in the present bcc Ti-Mo-Fe alloy deformed to tensile loading, we can expect that the HDDWs nucleated at twin tips tend to be aligned to the twin habit plane traces.

The occurrence of interrupted growth twins in the grain interiors of the present multilayered Ti-10Mo-xFe (x = 1–3) alloy can play a significant role on the mechanical behavior due to the development of localized stress gradients that are typically formed around twin tips. These in-grain stress concentrations may result in a high activity of dislocation plasticity, nucleation of twins or even lead to intragranular failure according to the underlying stress relaxation mechanism.[21,26,29,55,56]. In the present case, these stress concentrations are relaxed by dislocation plasticity on {112}<111> and {110}<111> slip systems, and {332}<113> twinning. In particular, our observations reveal that the accommodation of twin tips is mainly performed by dislocation plasticity, as reflected by the low density of twin plates around the twin tips and the high dislocation activity, mainly in the form of HDDWs. Deformation twinning is then not a relevant relaxation mechanism in the present multilayered material. We conclude that the excellent plastic accommodation of the twin tips has significant contribution to the good ductility of the multilayered Ti-Mo-Fe alloy (elongation of 28%).

## 5. Conclusions

We have investigated the propagation of {332}<113> twins in a multilayered Ti-10Mo-xFe (x = 1–3) alloy fabricated by multi-pass hot rolling. The material contains a macroscopic Fe-graded structure (about 130 μm width) between 1 and 3 wt% Fe in the direction perpendicular to rolling. The following conclusions can be drawn:

The Fe-graded structure has a significant influence on the twin propagation behavior. The propagation of {332}<113> twins that are nucleated in Fe-lean regions (~1 wt% Fe) is interrupted in the grain interiors at a specific Fe content, namely, about 2 wt% Fe. We ascribe this effect to the strong influence of Fe content on the stress for twin propagation, which has been experimentally validated.

Stress accommodation of the twin tips is mainly performed by dislocation plasticity on {112}<111> and {110}<111> slip systems. Deformation twinning is not a relevant relaxation mechanism of the stress concentrations around the twin tips in the present multilayered material.

The high dislocation activity around the twin tips leads to the formation of HDDWs, which are typically associated to strain localization phenomena. These dislocation configurations nucleate at the edges of the twin tips and propagate on highly stressed crystallographic planes. These dislocation configurations are not stable and evolve into loose dislocation arrangements. We ascribe this effect to the stress field around the twin tips. Due to the bcc structure and the deformation texture, HDDWs tend to be aligned to the twin habit plane traces.

The excellent plastic accommodation of the interrupted twin tips in the grain interiors results in the good ductility (total elongation of 28%) of the present multilayered Ti-10Mo-xFe alloy (x = 1–3).

## References

[CIT0001] Niinomi M (2008). Mechanical biocompatibilities of titanium alloys for biomedical applications. J Mech Beh Biom Met.

[CIT0002] Al-Zain Y, Sato Y, Kim HY, Hosoda H, Nam TH, Miyazaki S (2012). Room temperature aging behavior of Ti-Nb-Mo-based superelastic alloys. Acta Mater.

[CIT0003] Al-Zain Y, Kim HY, Hosoda H, Nam TH, Miyazaki S (2010). Shape memory properties of Ti-Nb-Mo biomedical alloys. Acta Mater.

[CIT0004] Geetha M, Singh AK, Asokamani R, Gogia AK (2009). Ti based biomaterials, the ultimate choice for orthopaedic implants – a review. Prog Mater Sci.

[CIT0005] Banerjee D, Williams JC (2013). Perspectives on titanium science and technology. Acta Mater.

[CIT0006] Clément N, Lenain A, Jacques PJ (2007). Mechanical property optimization via microstructural control of new metastable beta titanium alloys. JOM.

[CIT0007] Min XH, Emura S, Meng FQ, Mi GB, Tsuchiya K (2015). Mechanical twinning and dislocation slip multilayered dislocation microstructures in b-type Ti-Mo base alloy. Scripta Mater.

[CIT0008] Hida M, Sukedai E, Henmi C, Sakaue K, Terauchi H (1982). Stress induced products and ductility due to lattice instability of β phase single crystals of Ti-Mo alloys. Acta Metall.

[CIT0009] Ohyama H, Nishimura T (1995). Effects of alloying elements on deformation mode in Ti-V based β titanium alloy system. ISIJ Int.

[CIT0010] Ling FW, Starke EA, Lefevre BG (1975). Deformation behavior and texture development in beta Ti-V alloys. Metall Trans.

[CIT0011] Hanada S, Takemura A, Izumi O (1982). The mode of plastic deformation of β Ti-V alloys. Trans Jpn Inst Met.

[CIT0012] Christian JW (1983). Some surprising features of the plastic deformation of body-centered cubic metals and alloys. Metall Trans A.

[CIT0013] Taylor G (1992). Thermally-activated deformation of BCC metals and alloys. Prog Mater Sci.

[CIT0014] Lee SH, Hagihara K, Nakano T (2012). Microstructural and orientation dependence of the plastic deformation behavior in β-type Ti-15Mo-5Zr-3Al alloy single crystals. Metall Mater Trans A.

[CIT0015] Gysler A, Lütjering G, Gerold V (1974). Deformation behavior of age-hardened Ti-Mo alloys. Acta Metall.

[CIT0016] Kawabata T, Kawasaki S, Izumi O (1998). Mechanical properties of TiNbTa single crystals at cryogenic temperatures. Acta Mater.

[CIT0017] Tobe H, Kim HY, Inamura T, Hosoda H, Miyazaki S (2014). Origin of 332 twinning in metastable β-Ti alloys. Acta Mater.

[CIT0018] Rusakov GM, Litvinov AV, Litvinov VS (2006). Deformation twinning of titanium β-alloys of transition class. Met Sci Heat Treat.

[CIT0019] Bilby BA, Crocker AG (1965). The theory of the crystallography of deformation twinning. Proc R Soc A.

[CIT0020] Cottrell AH, Bilby BA (1951). A mechanism for the growth of deformation twins in crystals. Phil Mag.

[CIT0021] Christian JW, Mahajan S (1995). Deformation twinning. Prog Mater Sci.

[CIT0022] Steinmetz DR, Jäpel T, Wietbrock B, Eisenlohr P, Gutierrez-Urrutia I, Saeed-Akbari A, Hickel T, Roters F, Raabe D (2013). Revealing the strain hardening behavior of twinning induced plasticity steels through a dislocation density- and twin evolution-based constitutive model: theory, simulations, experiments. Acta Mater.

[CIT0023] Gutierrez-Urrutia I, Zaefferer S, Raabe D. (2010). The effect of grain size and grain orientation on deformation twinning in a Fe–22 wt.% Mn–0.6 wt.% C TWIP steel. Mater Sci Eng A.

[CIT0024] Fernandez A, Jerusalem A, Gutierrez-Urrutia I, Perez-Prado MT (2013). Three-dimensional investigation of grain boundary–twin interactions in a Mg AZ31 alloy by electron backscatter diffraction and continuum modeling. Acta Mater.

[CIT0025] Mahajan S (1981). Accommodation at deformation twins in bcc crystals. Metall Trans A.

[CIT0026] Eisenlohr A, Gutierrez-Urrutia I, Raabe D (2012). Adiabatic temperature increase associated with deformation twinning and dislocation plasticity. Acta Mater.

[CIT0027] Hull D (1961). Effect of grain size and temperature in slip, twinning and fracture in 3% silicon iron. Acta Metall.

[CIT0028] Worthington PJ, Smith E (1966). Slip, twinning and fracture in polycrystalline 3% silicon iron. Acta Metall.

[CIT0029] Mahajan S, Williams DF (1973). Deformation twinning in metals and alloys. Int Metall Rev.

[CIT0030] Meyers MA, Vöhringer O, Lubarda VA (2001). The onset of twinning in metals: A constitutive description. Acta Mater.

[CIT0031] Min XH, Tsuzaki K, Emura S, Tsuchiya K (2011). Enhancement of uniform elongation in high strength Ti-Mo based alloys by combination of deformation modes. Mater Sci Eng A.

[CIT0032] Gutierrez-Urrutia I, Zaefferer S, Raabe D (2009). Electron channeling contrast imaging of twins and dislocations in twinning-induced plasticity steels under controlled diffraction conditions in a scanning electron microscope. Scripta Mater.

[CIT0033] Gutierrez-Urrutia I, Zaefferer S, Raabe D (2013). Coupling of electron channeling with EBSD: toward the quantitative characterization of deformation structures in the SEM. JOM.

[CIT0034] Collings EW (1984). The physical metallurgy of titanium alloys.

[CIT0035] Kawabata T, Kawasaki S, Izumi O (1998). Mechanical properties of TiNbTa single crystals at cryogenic temperatures. Acta Mater.

[CIT0036] Hanada S, Izumi O (1980). Deformation of metastable beta Ti-15Mo-5Zr alloy single crystals. Metall Trans A.

[CIT0037] Sun F, Zhang JY, Marteleur M, Brozek C, Rauch EF, Veron M, Vermaut P, Jacques PJ, Prima F (2015). A new titanium alloy with a combination of high strength, high strain hardening and improved ductility. Scripta Mater.

[CIT0038] Hanada S, Ozeki M, Izumi O (1985). Deformation characteristics in β phase Ti-Nb alloys Metall Trans A.

[CIT0039] Gutierrez-Urrutia I, Raabe D (2012). Multistage strain hardening through dislocation substructure and twinning in a high strength and ductile weight-reduced Fe-Mn-Al-C steel. Acta Mater.

[CIT0040] Gutierrez-Urrutia I, Raabe D (2013). Microbanding mechanism in a Fe-22Mn-0.6C (wt.%) high-Mn twinning induced plasticity steel. Scripta Mater.

[CIT0041] Hughes DA (1993). Microstructural evolution in a non-cell forming metal: Al-Mg. Acta Metall Mater.

[CIT0042] Kuhlmann-Wilsdorf D (1989). Theory of plastic deformation:-properties of low energy dislocation structures. Mater Sci Eng A.

[CIT0043] Narita N, Takamura JI (1992). Deformation twinning in f.c.c. and b.c.c. metals. Nabarro FRN, editor. Dislocations in solids.

[CIT0044] Banerjee S, Naik UM (1996). Plastic instability in an omega forming Ti-15%Mo alloy. Acta Mater.

[CIT0045] Chen QZ, Ngan AHW, Duggan BJ (2003). Microstructure evolution in an interstitial-free steel during cold rolling at low strain levels. Proc R Soc Lond A.

[CIT0046] Chen QZ, Duggan BJ (2004). On cells and microbands formed in an interstitial-free steel during cold rolling at low to medium reductions. Metall Mater Trans A.

[CIT0047] Shen K, Duggan BJ (2007). Microbands and crystal orientation metastability in cold rolled interstitial-free steel. Acta Mater.

[CIT0048] Poddar D, Cizek P, Beladi H, Hodgson PD (2015). The evolution of microbands and their interaction with NbC precipitates during hot deformation of a Fe-30Ni-Nb model austenitic steel. Acta Mater.

[CIT0049] Hu SY, Henager CH, Chen LQ (2010). Simulations of stress-induced twinning and de-twinning: A phase field model. Acta Mater.

[CIT0050] Kamat SV, Hirth JP, Müllner P (1996). The effect of stress on the shape of a blocked deformation twin. Phil Mag A.

[CIT0051] Barnett M, Setty M, Siska F (2013). Estimating critical stresses required for twin growth in a magnesium alloy. Metall Mater Trans A.

[CIT0052] Nabarro FRN (1967). Theory of crystal dislocations.

[CIT0053] Hirth JP, Lothe L (1968). Theory of dislocations.

[CIT0054] Hosford WF (2010). Mechanical behavior of materials.

[CIT0055] Priestner R, Leslie WC (1965). Nucleation of deformation twins at slip plane intersections in B.C.C. metals. Phil Mag.

[CIT0056] Bieler TR, Eisenlohr P, Zhang C, Phukan HJ, Crimp MA (2014). Grain boundaries and interfaces in slip transfer. Curr Opin Solid State Mater Sci.

